# Hsa_circ_0000231 knockdown inhibits the glycolysis and progression of colorectal cancer cells by regulating miR-502-5p/MYO6 axis

**DOI:** 10.1186/s12957-020-02033-0

**Published:** 2020-09-29

**Authors:** Yanhe Liu, Hui Li, Xiaoyi Ye, Anlong Ji, Xiangwei Fu, Haishan Wu, Xiangyong Zeng

**Affiliations:** 1 Department of General Surgery, The Second Affiliated Hospital of Hainan Medical University, No. 48 Baishuitang Road, Haikou, 570311 Hainan Province China; 2 Department of Geriatrics, The Second Affiliated Hospital of Hainan Medical University, Haikou City, Hainan Province China

**Keywords:** Circular RNAs, Hsa_circ_0000231, MiR-502-5p, MYO6, Colorectal cancer

## Abstract

**Background:**

Colorectal cancer (CRC) poses a heavy threat to human health owing to its high incidence and mortality. Circular RNAs (circRNAs) were investigated to participate in the progression of CRC, whereas there was no revenant data on the CRC process regulated by hsa_circ_0000231. This study aimed to explore the effects of hsa_circ_0000231 on CRC progression and underneath regulatory mechanism.

**Methods:**

The expression levels of hsa_circ_0000231, miR-502-5p, and Myosin VI (MYO6) mRNA were detected by quantitative real time polymerase chain reaction (qRT-PCR). Western blot was employed to determine the protein expression levels of MYO6 and proliferating cell nuclear antigen (PCNA). The effects of hsa_circ_0000231 on cell proliferation, apoptosis, migration, and invasive in CRC were determined by cell counting kit-8 proliferation (CCK-8) and colony formation assays, flow cytometry analysis, wound-healing assay, and transwell invasion assay, respectively. Glucose uptake and lactate production were severally illustrated by glucose assay kit and lactate assay kit. The relationship between miR-502-5p and hsa_circ_0000231 or MYO6 was predicted by circular RNA interactome or targetScan online databases, and identified by dual-luciferase reporter and RNA immunoprecipitation (RIP) assays. In vivo tumor formation assay was carried out to determine the effects of hsa_circ_0000231 knockdown on tumor growth in vivo.

**Results:**

Hsa_circ_0000231 expression was dramatically upregulated while miR-502-5p was obviously downregulated in CRC tissues and cells compared with control groups. Hsa_circ_0000231 knockdown repressed the expression levels of MYO6 and PCNA protein. Functionally, hsa_circ_0000231 knockdown repressed cell glycolysis, proliferation, migration and invasion, and induced cell apoptosis, whereas these effects were decreased by miR-502-5p inhibitor. Mechanistically, hsa_circ_0000231 acted as a sponge of miR-502-5p and miR-502-5p bound to MYO6. Furthermore, hsa_circ_0000231 knockdown decreased tumor volume and weight of CRC in vivo.

**Conclusion:**

Hsa_circ_0000231 knockdown inhibited CRC progression and glycolysis by downregulating MYO6 expression through sponging miR-502-5p, which might provide a theoretical basis in further studying circ_0000231-directed therapy in CRC.

## Introduction

Colorectal cancer (CRC) is one of the main reasons of cancer-caused mortality worldwide [1]. The survival proportion of patients is also low owing to CRC distant metastasis with a 5-year survival rate is only about 10% [2]. Although the progress of diagnosis and therapy for CRC has been developed, the morbidity of CRC is still high in elderly people. Therefore, deeply understanding the pathogenesis of the CRC process is necessary to its therapy.

Circular RNAs (circRNAs) are a kind of non-coding RNA and are more stable than their linear form with closed loop [3]. CircRNAs are expressed in various tissues and organs, and widely take part in various cancer developments, including CRC [4, 5]. For instance, circRNA CBL.11 was studied to inhibit cell proliferation in CRC [6]. Hsa_circ_0000523 silencing suppressed cell proliferation and induced apoptosis in CRC [7]. Additionally, Li et al. revealed circ_DDX17 was weakly expressed and its silencing accelerated the proliferation and metastasis of CRC cells [4]. However, there is no data on the regulatory mechanism of CRC by hsa_circ_0000231.

MicroRNAs (miRNAs) are a kind of transcripts of 18-22 nucleotides and work via targeting mRNA 3′-untranslated regions (3′UTR) [8]. MiR-502-5p is indicated to participate in cancer progression [9–11]. For example, miR-502-5p overexpression represses cell metastasis in bladder cancer [12]. MiR-502-5p is also indicated to regulate the tumorigenesis and metastasis of gastric cancer [13]. Nevertheless, the data on the impacts of miR-502-5p regulating CRC progression is little.

Myosin VI (MYO6) is a member of the actin-related myosin family and takes part in multiply vital biologic processes [14]. It was reported that MYO6 was related to contribute to the development of human cancers [14, 15]. Lei et al. indicated that miR-143 and miR-145 suppressed cell metastasis via targeting MYO6 in gastric cancer [16]. Xu et al. revealed that MYO6 promoted cell proliferation in human glioma cells [15]. MYO6 was overexpressed in various cancers, which suggested that it could be used as a biomarker for cancer treatment.

In this study, hsa_circ_0000231 expression and MYO6 protein expression were dramatically increased while miR-502-5p expression was obviously decreased in CRC tissues and cells. Hsa_circ_0000231 regulated cell proliferation, migration, invasion, apoptosis, and glycolysis by controlling MYO6 expression through binding to miR-502-5p in CRC. Furthermore, hsa_circ_0000231 knockdown repressed tumor formation in vivo.

## Materials and methods

### Sample and cell culture

The Ethics Committee of the Second Affiliated Hospital of Hainan Medical University approved this experiment. Forty pairs of CRC tissues and paracancerous healthy tissues were collected from the Second Affiliated Hospital of Hainan Medical University. Tissues were kept in liquid nitrogen. All patients signed the written informed consents.

The Otwo Biotech (Shenzhen, China) supplied human normal colonic epithelial cells NCM460 and human CRC cell lines HCT116 and LoVo. Cells were cultivated in Dulbecco’s modified Eagle’s medium (DMEM; Invitrogen, Carlsbad, CA, USA) or Roswell Park Memorial Institute-1640 (RPMI-1640) with 10% fetal bovine serum (FBS) and 1% streptomycin/penicillin (Invitrogen) at 37 °C with 5% CO_2_.

### Cell transfection

Small interfering RNAs targeting hsa_circ_0000231 (si-hsa_circ_0000231#1 and si-hsa_circ_0000231#2), short hairpin RNA (shRNA) against hsa_circ_0000231 (sh-hsa_circ_0000231), miR-502-5p mimic (miR-502-5p), hsa_circ_0000231 overexpression vector (hsa_circ_0000231), miR-502-5p inhibitor (anti-miR-502-5p), MYO6 overexpression vector (MYO6), and their control groups (si-NC, sh-NC, NC, circ-NC, anti-NC, and vector) were amplified by Ribobio Co., Ltd. (Guangzhou, China). Cells were transfected with various treatments with Lipofectamine 2000 in agreement with literature methods [17] and collected at defined time. The sequences used in this part were listed in Supplementary Table 1.

### RNA extraction and quantitative real-time polymerase chain reaction

The sample was lysed with TransZol (TransGen, Beijing, China), and RNA concentration was measured by the NanoDrop-1000 instrument (Thermo Fisher, Waltham, MA, USA). Then cDNA was synthesized with a reverse transcription kit (Takara, Dalian, China). After that, qRT-PCR detection kit (Takara) was employed to quantify the expression of circRNA, miRNA, or mRNA. GAPDH and U6 were chosen as references. Data was calculated by the 2^−ΔΔCt^ method. The sense and anti-sense primers were presented in Supplementary Table 1.

### RNase R treatment assay

The RNA of HCT116 and LoVo cells was extracted and incubated with RNase R (Sigma, St Louis, MO, USA). Then, the samples were purified and results were detected with RT-PCR.

### Cell counting kit-8 assay

Cell viability was detected with a CCK-8 kit (Beyotime, Jiangsu, China). Briefly, HCT116 and LoVo cells were calculated and seeded in a 96-well plate (5 × 10^3^ per well) and cultured for 24 h. Then si-hsa_circ_0000231#1, si-hsa_circ_0000231#2, anti-miR-502-5p, miR-502-5p, or MYO6 was transfected into cells with their controls, and cells were continued to cultivate for 24, 48, and 72 h. Following 10 μL CCK-8 regent (Beyotime) was added into plate, and cells were continued to culture another 4 h. Results were analyzed by detecting the absorbance at a wavelength of 450 nm using a microplate reader (Thermo Fisher).

### Colony formation assay

The proliferative ability of HCT116 and LoVo cells was detected by colony formation assay. In short, HCT116 and LoVo cells were cultivated in a 6-well plate for 14 h. And si-hsa_circ_0000231#1, si-hsa_circ_0000231#2, anti-miR-502-5p, miR-502-5p, or MYO6 was transfected into cells with their controls. Two weeks later, the medium was removed and the proliferating colonies were fixed with paraformaldehyde. After that, cells were incubated with 1% crystal violet. Results were analyzed by counting colonies number. A colony was defined when its cell number exceeded 50.

### Flow cytometry analysis

HCT116 and LoVo cells grown at logarithmic period were collected and washed using phosphate buffer solution (PBS). Then cells were suspended with binding buffer, and were incubated with Annexin V-FITC for 5 min and propidium iodide (KeyGen Biotech, Nanjing, China) for 30 min. Cell apoptosis was determined by a FACSort flow cytometer (BD Biosciences, San Diego, CA, USA).

### Cleaved Caspase-3 (C-caspase-3) activity assay

C-caspase-3 activity was detected with Caspase 3 Activity Assay Kit (Beyotime,) according to the manufacturer’s protocol. In short, HCT116 and LoVo cells were collected, washed with PBS, and lysed. The lysates were incubated with C-caspase-3 reaction reagent. The results were visualized by a plate-reading luminometer (Thermo Fisher).

### Transwell invasion assay

HCT116 and LoVo cells (1 × 10^5^) were cultivated in 12-well upper chamber (8-mm pore; Corning, Shanghai, China) with Matrigel (100 μL; Corning) with serum-free medium to examine cell invasion. Medium containing 20% FBS was added into the lower chamber. Chambers were taken out from the plate after 24 h and cells were washed using PBS. Then, cells were incubated with methanol and crystal violet. Invaded cells were counted with a microscope at a 100 magnification.

### Wound-healing assay

The migration ability of HCT116 and LoVo cells was determined by the wound-healing assay. In short, HCT116 and LoVo cells were grown in a 6-well plate. The wounds were created with a pipette tip when cells approached 100% confluence. Then medium without FBS was added into plate. Following cells were cultivated for 24 h. The results were determined by calculating the area occupied by migrated cells with a microscope with a 100 magnification.

### Glucose uptake and lactate production assay

The glucose and lactate levels were detected using Glucose Assay Kit (Abcam, Cambridge, UK) and Lactate Assay Kit (Abcam) according to the protocols, respectively. Briefly, HCT116 and LoVo cells were cultivated in a 6-well plate for 48 h. Then, supernatants were collected. And glucose uptake and lactate production were analyzed by detecting absorbance at 450 nm or 570 nm using a microplate reader (Thermo Fisher).

### Western blot analysis

HCT116 and LoVo cells were lysed. The sample was loaded into 12% SDS-PAGE. After that, proteins bands were transferred onto nitrocellulose membranes. Following bands were blocked in 5% skim milk. Then, membranes were incubated with primary antibodies. Following, a secondary antibody labeled with horseradish peroxidase was utilized to incubate membranes. Results were visualized by enhanced chemiluminescence (Pierce, Rockford, IL, USA). GAPDH was chosen as a control. The primary antibodies were anti-hexokinase 2 (anti-HK2) (1:1000; Abcam), anti-MYO6 (1:500; Abcam), anti-proliferating cell nuclear antigen (anti-PCNA) (1:1000; Abcam), and anti-GAPDH (1:2500; Abcam).

### Dual-luciferase reporter assay

The binding sequences between miR-502-5p and hsa_circ_0000231 or MYO6 were predicted by circular RNA interactome or targetScan online database. The wide-type (wt) sequences of hsa_circ_0000231 and MYO6 3′UTR contained the target sequences of miR-502-5p were synthesized and inserted into the pmirGLO vector (Promega, Madison, WI, USA). The sequences bound to miR-502-5p in hsa_circ_0000231 and MYO6 3′UTR were mutated, and the mutant (mut) hsa_circ_0000231 and MYO6 3′UTR were synthesized and sub-cloned into pmirGLO vector (Promega). Then plasmids were co-transfected into cells with miR-502-5p or NC and cells were cultured for 48 h. Luciferase activities were detected by dual-luciferase reporter assay kit (Promega). *Renilla* luciferase activity was chosen as a reference.

### RIP assay

HCT116 and LoVo cells were collected and lysed. Cell lysates were incubated with magnetic beads coated with anti-Ago2 (Bioss, Beijing, China) or anti-IgG (Abcam) for 24 h. RNA was purified, and the enrichment of hsa_circ_0000231, miR-502-5p, and MYO6 was detected by qRT-PCR.

### In vivo tumor formation assay

All protocols were approved by the Animal Care Committee of the Second Affiliated Hospital of Hainan Medical University. Charles River (Beijing, China) provided nude mice. HCT116 (5 × 10^6^) cells transfected with sh-hsa_circ_0000231 or sh-NC were injected into the flank of mice. Tumors volume was measured every 5 days. All mice were euthanized after 30 days. The volume and weight of tumors were analyzed.

### Statistical analysis

Figures were made using the GraphPad Prism version 5.0 and image J software. Data were shown as means ± standard deviations based on three replicates. Significant differences were compared by one-way analysis of variance. *P* < 0.05 was considered statistically significant.

## Results

### Hsa_circ_0000231 is upregulated in CRC tissues and cells with poor survival rate

**Fig. 1:**
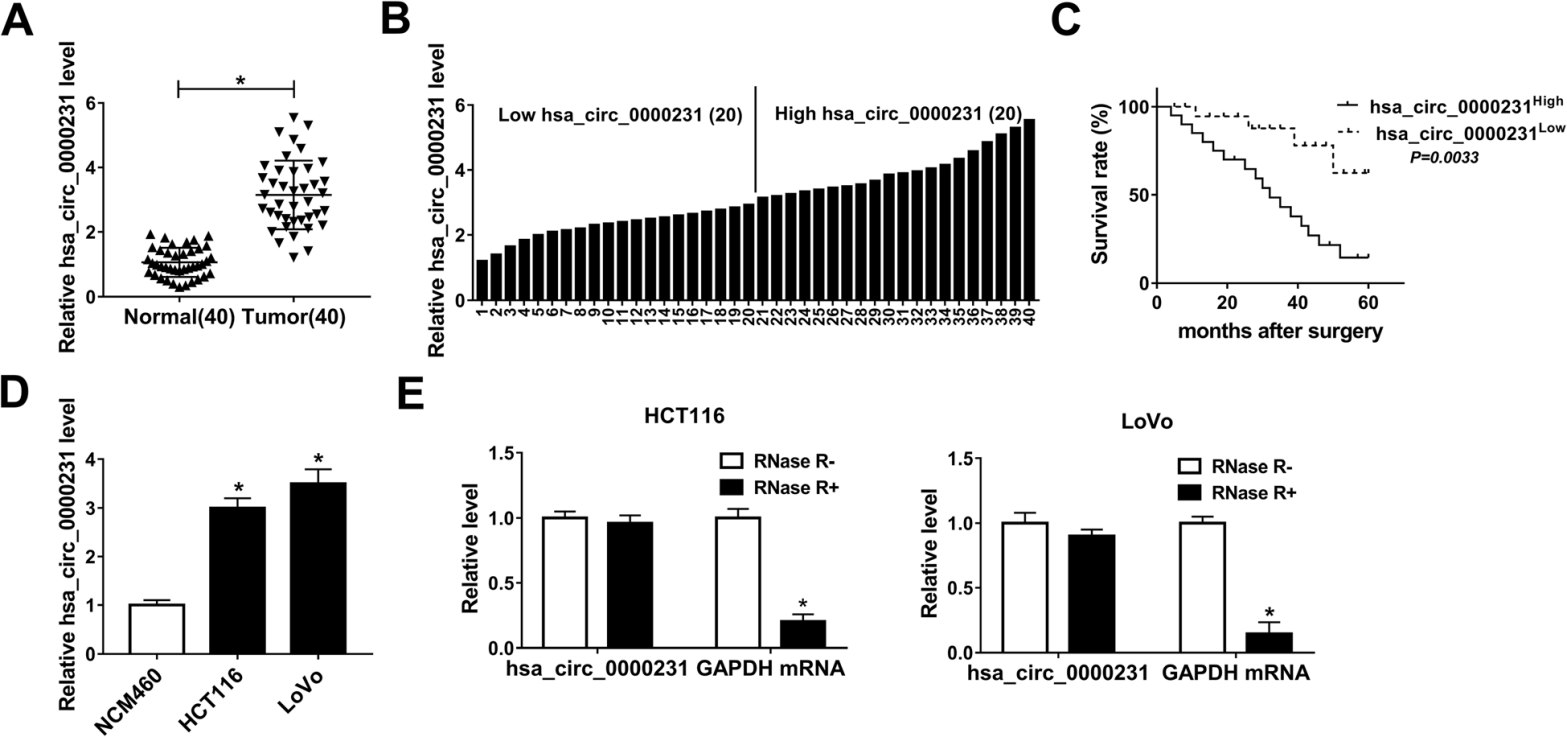
Hsa_circ_0000231 is overexpressed in CRC tissues and cells with a low survival rate. **a** QRT-PCR results revealed that the expression level of hsa_circ_0000231 was dramatically upregulated in CRC tissues compared with adjacent normal tissues. **b** Forty pairs of CRC tissues were divided into two groups based on hsa_circ_0000231 expression level. **c** Kaplan-Meier analysis showed that hsa_circ_0000231 expression level was negatively related to survival rate. **d** The expression of hsa_circ_0000231 was significantly increased in HCT116 and LoVo cells relative to NCM460 cells. **e** RNase R treatment assay revealed that hsa_circ_0000231 was a circular RNA. *P < 0.05

In order to study the role of hsa_circ_0000231 in CRC, hsa_circ_0000231 expression level was detected by qRT-PCR in 40 pairs of CRC tissues and adjacent normal tissues. Results showed that the expression of hsa_circ_0000231 was dramatically upregulated in CRC tissues relative to normal tissues (Fig. 1a). Meanwhile, qRT-PCR results explained that hsa_circ_0000231 expression was higher in HCT116 and LoVo cells than that in NCM460 cells (Fig. 1d). Further, the 40 CRC tissues were divided into two groups (20 hsa_circ_0000231 higher expression group and 20 hsa_circ_0000231 lower expression group) based on hsa_circ_0000231 expression level (Fig. 1b). The clinical role of hsa_circ_0000231 was analyzed and results showed that hsa_circ_0000231 high expression was related to low survival rate (Fig. 1c). In order to illustrate whether hsa_circ_0000231 was a circular RNA, the hsa_circ_0000231 RNA derived from HCT116 and LoVo cells was treated with RNase R. Results showed that hsa_circ_0000231 was more stable than GAPDH mRNA (Fig. 1e). These data implicated that hsa_circ_0000231 played an important role in CRC progression.

### Hsa_circ_0000231 knockdown inhibits glycolysis, cell proliferation, migration, and invasion, whereas induces cell apoptosis in CRC

**Fig. 2:**
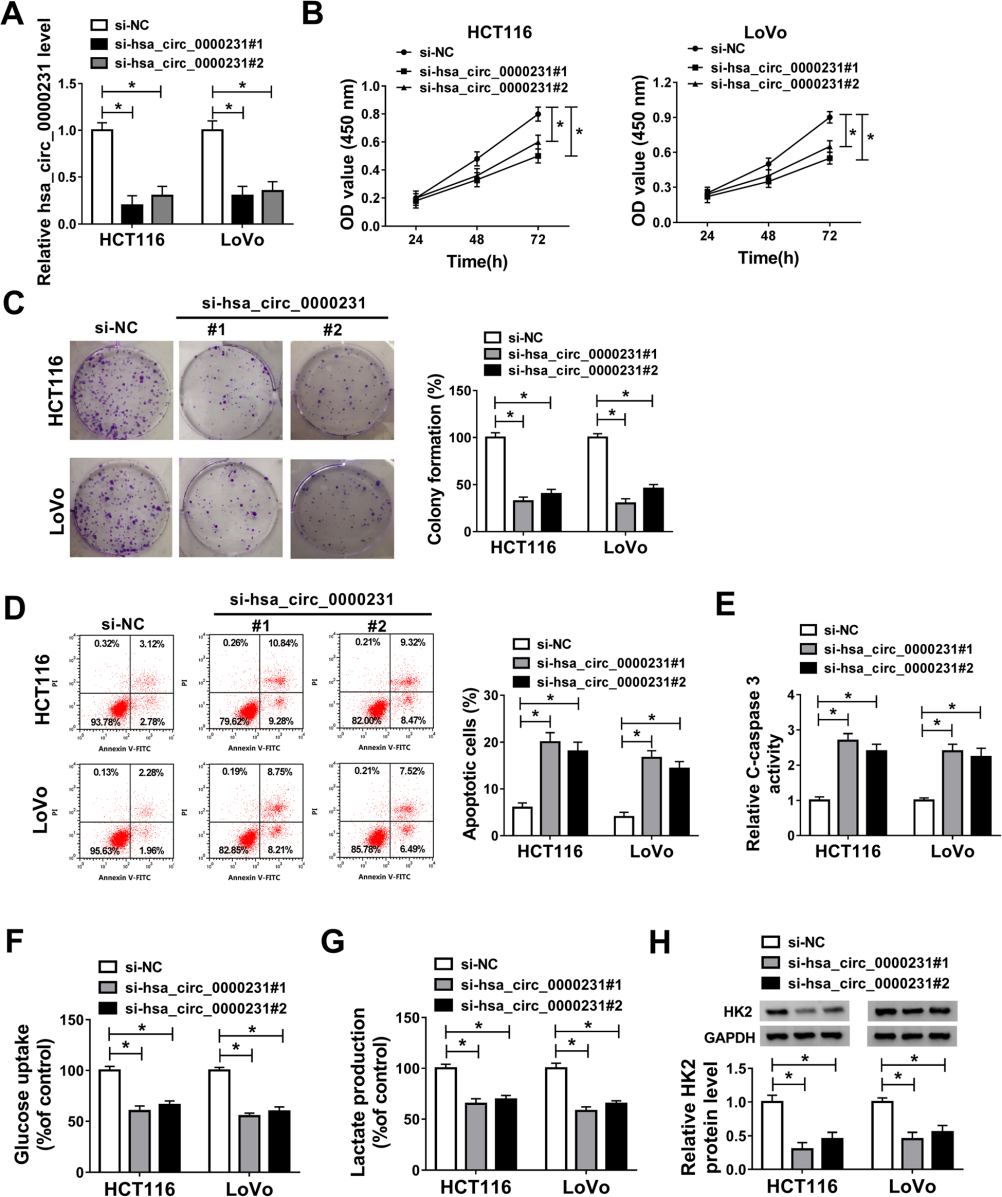
Hsa_circ_0000231 knockdown inhibits cell glycolysis and proliferation, and induced cell apoptosis in CRC. **a** Hsa_circ_0000231 silencing dramatically repressed hsa_circ_0000231 expression in HCT116 and LoVo cells. **b** and **c** CCK-8 and colony formation assays revealed that cell proliferation was suppressed by hsa_circ_0000231 silencing in both HCT116 and LoVo cells. **d** and **e** Flow cytometry and C-caspase-3 activity assays showed that hsa_circ_0000231 knockdown induced the apoptosis of HCT116 and LoVo cells. **f** and **g** Glucose uptake and lactate production assays were employed to severally explain hsa_circ_0000231 knockdown inhibited glucose uptake and lactate production in both HCT116 and LoVo cells. **h** Western blot analysis showed that si-hsa_circ_0000231#1 and si-hsa_circ_0000231#2 obviously downregulated HK2 protein expression in both HCT116 and LoVo cells. *P < 0.05

In order to explore the functional characteristics of hsa_circ_0000231 in CRC development, the interfering efficiency of si-hsa_circ_0000231#1 and si-hsa_circ_0000231#2 was firstly detected by qRT-PCR. Results showed that the expression level of hsa_circ_0000231 was greatly decreased after si-hsa_circ_0000231#1 and si-hsa_circ_0000231#2 transfection (Fig. 2a). Then the effects of hsa_circ_0000231 silencing on CRC progression were studied. CCK-8 and colony formation assays explained that cell viability and colony-forming ability were repressed by hsa_circ_0000231 knockdown, respectively, in both HCT116 and LoVo cells (Fig. 2b and c). Flow cytometry analysis showed that hsa_circ_0000231 knockdown promoted cell apoptosis in both HCT116 and LoVo cells (Fig. 2d). Meanwhile, C-caspase-3 activity assay revealed that C-caspase-3 activity was accelerated after hsa_circ_0000231#1 and si-hsa_circ_0000231#2 transfection in both HCT116 and LoVo cells (Fig. 2e). Transwell invasion and wound-healing assays demonstrated that cell invasion and migration abilities were hindered by hsa_circ_0000231 knockdown in both HCT116 and LoVo cells (Supplementary Figure 1A and B). Finally, the effects of hsa_circ_0000231 silencing on Warburg effect were explained. Data showed that glucose uptake and lactate production were lower in hsa_circ_0000231#1 and si-hsa_circ_0000231#2 groups than that in si-NC group (Fig. 2f and g). HK2 was indicated that it was a vital metabolic enzyme in glycolysis, and could promote glucose uptake [18]. Therefore, the effect of hsa_circ_0000231 silencing on HK2 expression was explored. Western blot results showed that hsa_circ_0000231 dramatically repressed HK2 protein expression (Fig. 2h). All these data explained that hsa_circ_0000231 knockdown repressed glycolysis, cell proliferation, migration, and invasion, whereas promoted cell apoptosis in CRC.

### Hsa_circ_0000231 directly associates with miR-502-5p in CRC cells

**Fig. 3:**
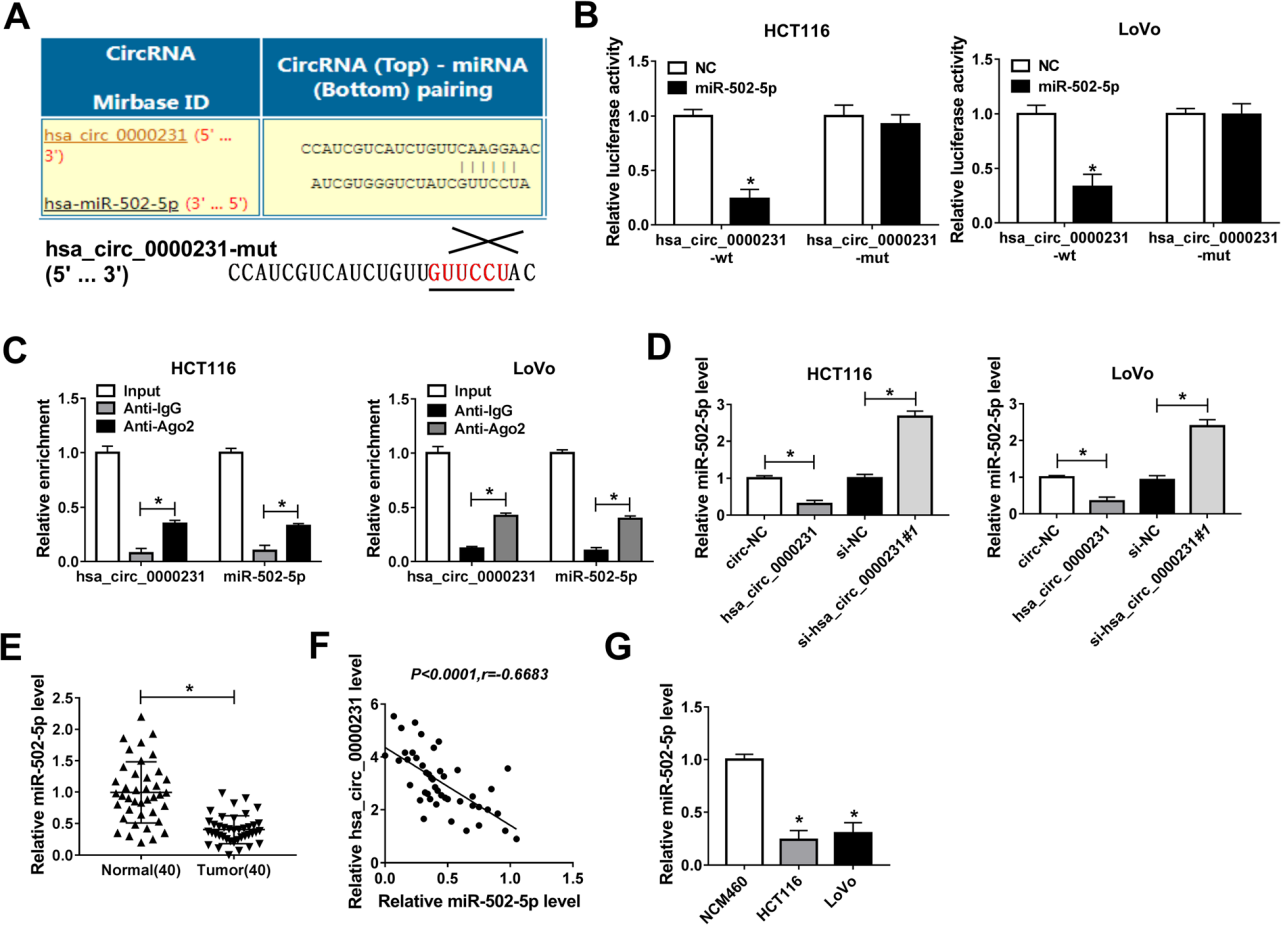
Hsa_circ_0000231 acts as a sponge of miR-502-5p. **a** Circular RNA interactome online database predicted the binding sites between hsa_circ_0000231 and miR-502-5p. **b** The predicted binding sequence of miR-502-5p in hsa_circ_0000231 was verified by dual-luciferase reporter assay in both HCT116 and LoVo cells. **c** RIP assay revealed that the direct association between hsa_circ_0000231 and miR-502-5p in both HCT116 and LoVo cells. **d** QRT-PCR explained hsa_circ_0000231 overexpression obviously downregulated miR-502-5p expression, and hsa_circ_0000231 knockdown apparently upregulated miR-502-5p expression in both HCT116 and LoVo cells. **e** and **g** The expression level of miR-502-5p was obviously decreased in CRC tissues and cells compared with control groups. **f** Hsa_circ_0000231 expression was negatively related to miR-502-5p expression. *P < 0.05

In order to study the underlying regulatory mechanism of hsa_circ_0000231 in CRC progression, the binding sites between hsa_circ_0000231 and miR-502-5p were predicted by a circular RNA interactome online database (Fig. 3a). The predicted target sites were verified by dual-luciferase reporter assay and results showed that the luciferase activity of hsa_circ_0000231-wt + miR-502-5p group was obviously decreased compared with that in hsa_circ_0000231-mut + miR-502-5p group in both HCT116 and LoVo cells (Fig. 3b). Meanwhile, RIP assay revealed that hsa_circ_0000231 and miR-502-5p were significantly enriched by anti-Ago2 rather than by anti-IgG in HCT116 and LoVo cells (Fig. 3c). Furthermore, the effects of hsa_circ_0000231 overexpression and knockdown on miR-502-5p expression were determined. QRT-PCR results showed that hsa_circ_0000231 overexpression inhibited miR-502-5p expression and hsa_circ_0000231 knockdown upregulated miR-502-5p expression level in HCT116 and LoVo cells (Fig. 3d). Besides, the expression level of miR-502-5p in CRC tissues and cells was detected by qRT-PCR, and results showed that miR-502-5p expression level was obviously downregulated in CRC tissues and cells compared with control groups (Fig. 3e and g). Pearson correlation analysis revealed that hsa_circ_0000231 was negatively correlated with miR-502-5p (Fig. 3f). These results showed that hsa_circ_0000231 functioned as a sponge of miR-502-5p in CRC cells.

### Hsa_circ_0000231 knockdown inhibits cell glycolysis and progression by sponging miR-502-5p in CRC

**Fig. 4:**
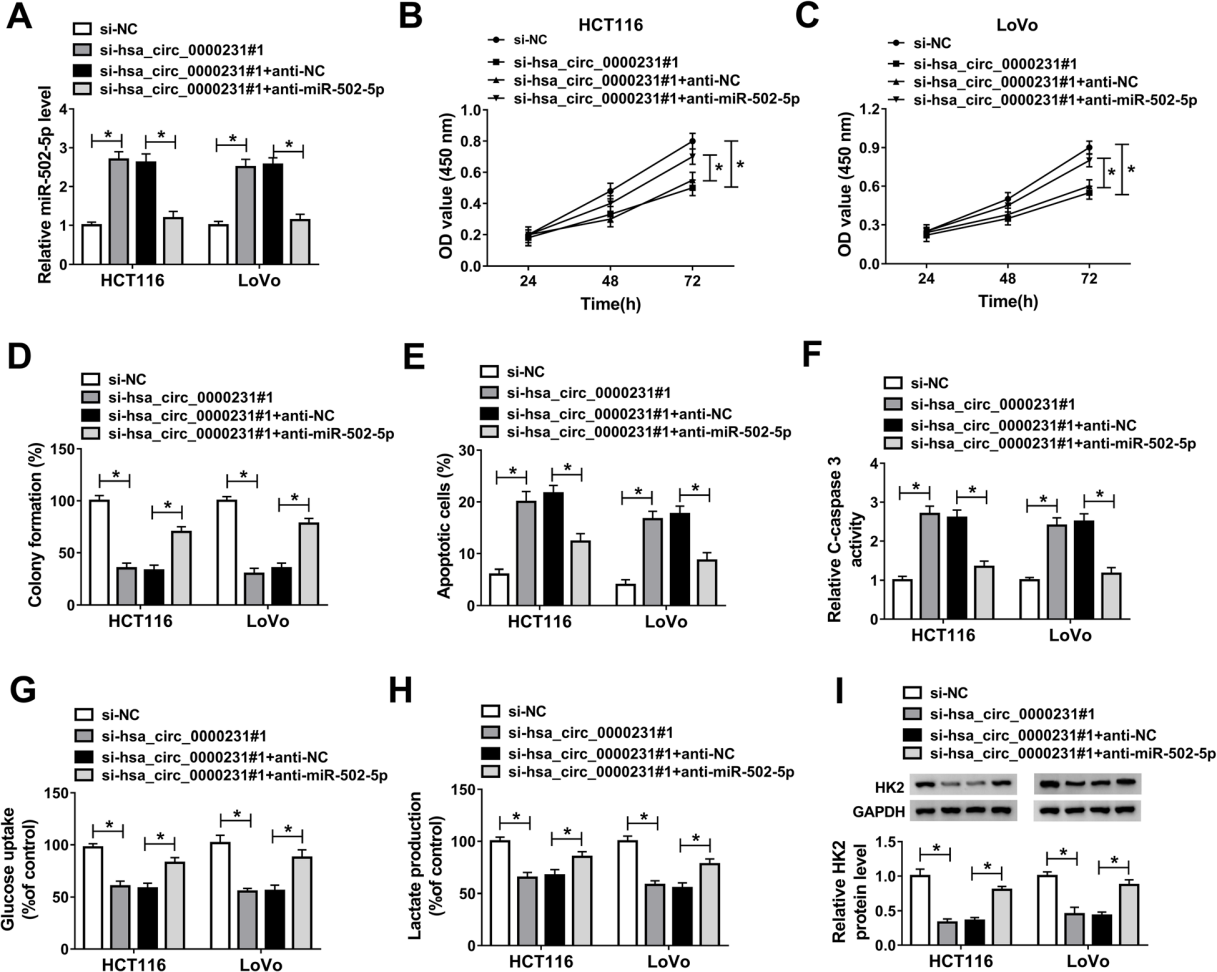
Hsa_circ_0000231 regulates the proliferation, glycolysis, and apoptosis of CRC cells by sponging miR-515-5p. **a** MiR-502-5p inhibitor restrained the promotion effects of hsa_circ_0000231 knockdown on miR-502-5p expression in HCT116 and LoVo cells. **b**-**d** MiR-502-5p inhibitor restrained the repression effect of hsa_circ_0000231 knockdown on the proliferation of HCT116 and LoVo cells. **e** MiR-502-5p inhibitor hindered the promotion impact of hsa_circ_0000231 knockdown on the apoptosis of HCT116 and LoVo cells. **f** MiR-502-5p inhibitor restrained the promotion effect of hsa_circ_0000231 silencing on C-caspase-3 activity in both HCT116 and LoVo cells. **g** and **h** MiR-502-5p inhibitor abolished the repression effectS of hsa_circ_0000231 knockdown on glucose uptake and lactate production in both HCT116 and LoVo cells. **i** MiR-502-5p inhibitor restrained the repression effect of hsa_circ_0000231 knockdown on HK2 protein expression in both HCT116 and LoVo cells. *P < 0.05

In order to study the functional effects between hsa_circ_0000231 and miR-502-5p on CRC progression, the impacts between hsa_circ_0000231 and miR-502-5p on miR-502-5p expression were firstly detected in both HCT116 and LoVo cells. QRT-PCR analysis showed that miR-502-5p expression level was dramatically upregulated by hsa_circ_0000231 knockdown and was decreased by miR-502-5p inhibitor (Fig. 4a). After that, the effects between hsa_circ_0000231 knockdown and miR-502-5p inhibitor on CRC progression were evaluated. CCK-8 and colony formation assays explained that hsa_circ_0000231 knockdown repressed cell proliferation in HCT116 and LoVo cells, whereas miR-502-5p inhibitor hindered this effect (Fig. 4b-d). Flow cytometry assay showed that hsa_circ_0000231 silencing promoted cell apoptosis in HCT116 and LoVo cells and this effect was partially attenuated by anti-miR-502-5p (Fig. 4e). Meanwhile, C-caspase-3 activity assay showed that C-caspase-3 activity was promoted by hsa_circ_0000231 silencing and this phenomenon was partially abolished by anti-miR-502-5p (Fig. 4f). Furthermore, transwell invasion and wound-healing assays revealed that cell invasion and migration abilities were hindered after si-hsa_circ_0000231#1 transfection; however, these effects were partially relieved by miR-502-5p inhibitor (Supplementary Figure 2A and B).

After that, the effects between hsa_circ_0000231 and miR-502-5p on glycolysis were studied in both HCT116 and LoVo cells. Results showed that hsa_circ_0000231 knockdown inhibited glucose uptake and lactate production, whereas these effects were decreased by miR-502-5p inhibitor (Fig. 4g and h). Western blot analysis showed that HK2 protein expression was repressed by hsa_circ_0000231 silencing and this phenomenon was hindered by miR-502-5p inhibitor (Fig. 4i). All these evidences showed that hsa_circ_0000231 knockdown repressed CRC progression and cell glycolysis by associating with miR-502-5p.

### Hsa_circ_0000231 regulates MYO6 expression by sponging miR-502-5p in CRC cells

**Fig. 5:**
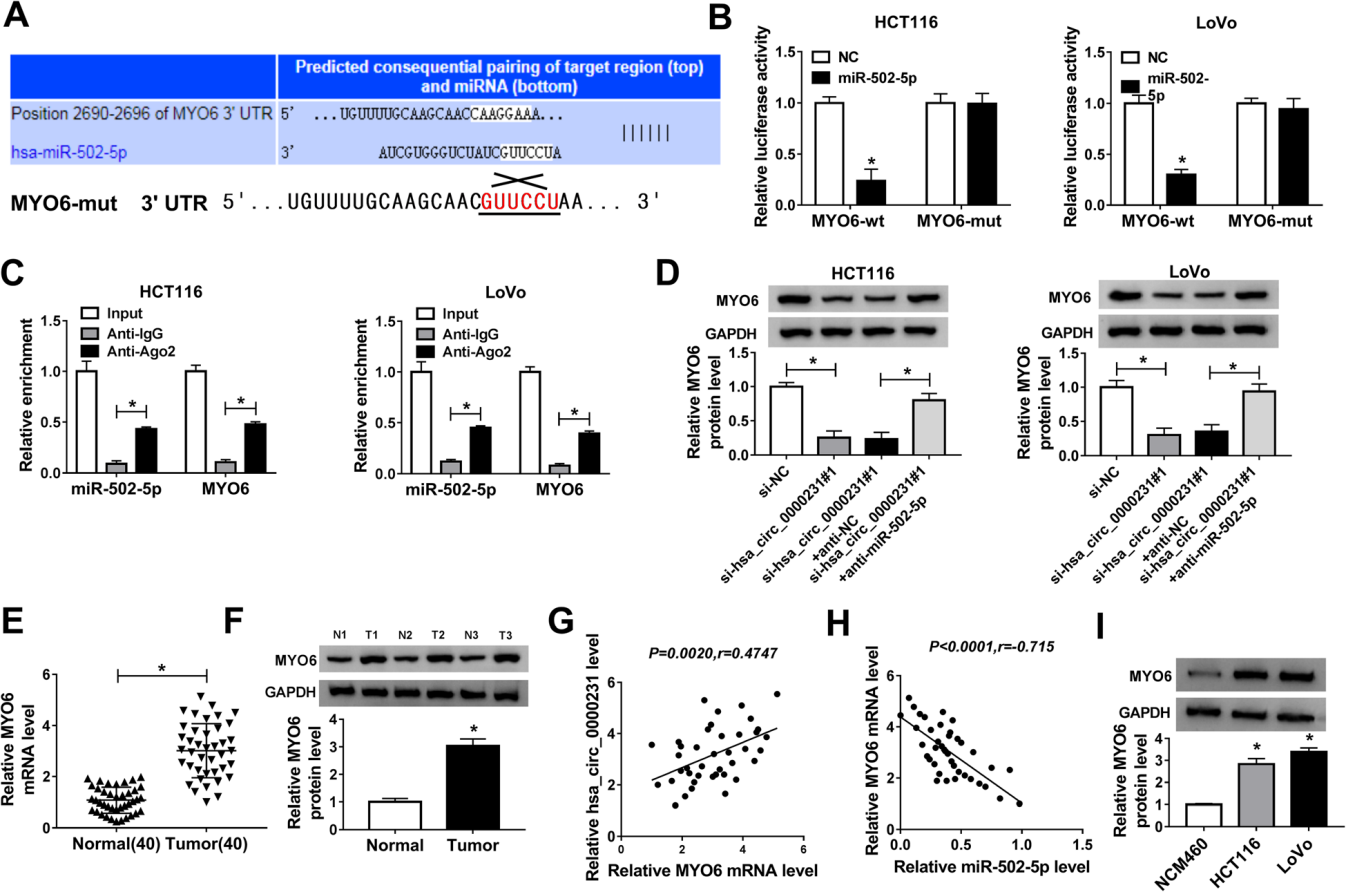
Hsa_circ_0000231 knockdown inhibits MYO6 expression by sponging miR-502-5p. **a** The binding sites between miR-502-5p and MY06 were predicted by targetScan online database. **b** The luciferase activities were detected by dual-luciferase reporter assay. **c** RIP assay was employed to explain the direct target between miR-502-5p and MYO6. **d** QRT-PCR explained that hsa_circ_0000231 regulated MYO6 expression by associating with miR-502-5p. **e** MYO6 mRNA expression was dramatically upregulated in CRC tissues compared with paracancerous normal tissues. **f** and **i** MYO6 protein expression level was significantly increased in CRC tissues and HCT116 and LoVo cells relative to control groups. **g** Pearson correlation analysis revealed that there was a poor relation between MYO6 level and hsa_circ_0000231 level. **h** MYO6 level was negatively correlated with miR-502-5p level. *P < 0.05

In order to further study the underneath regulatory mechanism of miR-502-5p in CRC progression, the target gene of miR-502-5p was predicted by targetScan online database (Fig. 5a). Dual-luciferase reporter assay explained that the luciferase activity of MYO6-wt + miR-502-5p group was dramatically decreased in HCT116 and LoVo cells, but the luciferase activity of MYO6-mut + miR-502-5p group was not obviously changed (Fig. 5b). RIP assay revealed that miR-502-5p and MYO6 were dramatically enriched in anti-Ago2 group compared with that in anti-IgG group (Fig. 5c). Then, the effects of hsa_circ_0000231 knockdown or miR-502-5p inhibitor on MYO protein expression were detected in HCT116 and LoVo cells, and results showed that hsa_circ_0000231 knockdown repressed MYO6 protein expression, whereas this effect was decreased by miR-502-5p inhibitor (Fig. 5d). After that, MYO6 mRNA expression was detected by qRT-PCR in CRC tissues, and results showed that MYO6 expression was dramatically upregulated compared with normal tissues (Fig. 5e). Western blot analysis showed that MYO6 protein expression was also upregulated in CRC tissues and cells relative to control groups (Fig. 5f and i). Furthermore, Pearson correlation analysis revealed that there was a poor relation between MYO6 and hsa_circ_0000231, while MYO6 was negatively correlated with miR-505-5p (Fig. 5g and h). These data revealed that hsa_circ_0000231 regulated MYO6 expression by associating with miR-502-5p in CRC cells.

### MiR-502-5p inhibits the biologic processes of CRC by targeting MYO6

**Fig. 6:**
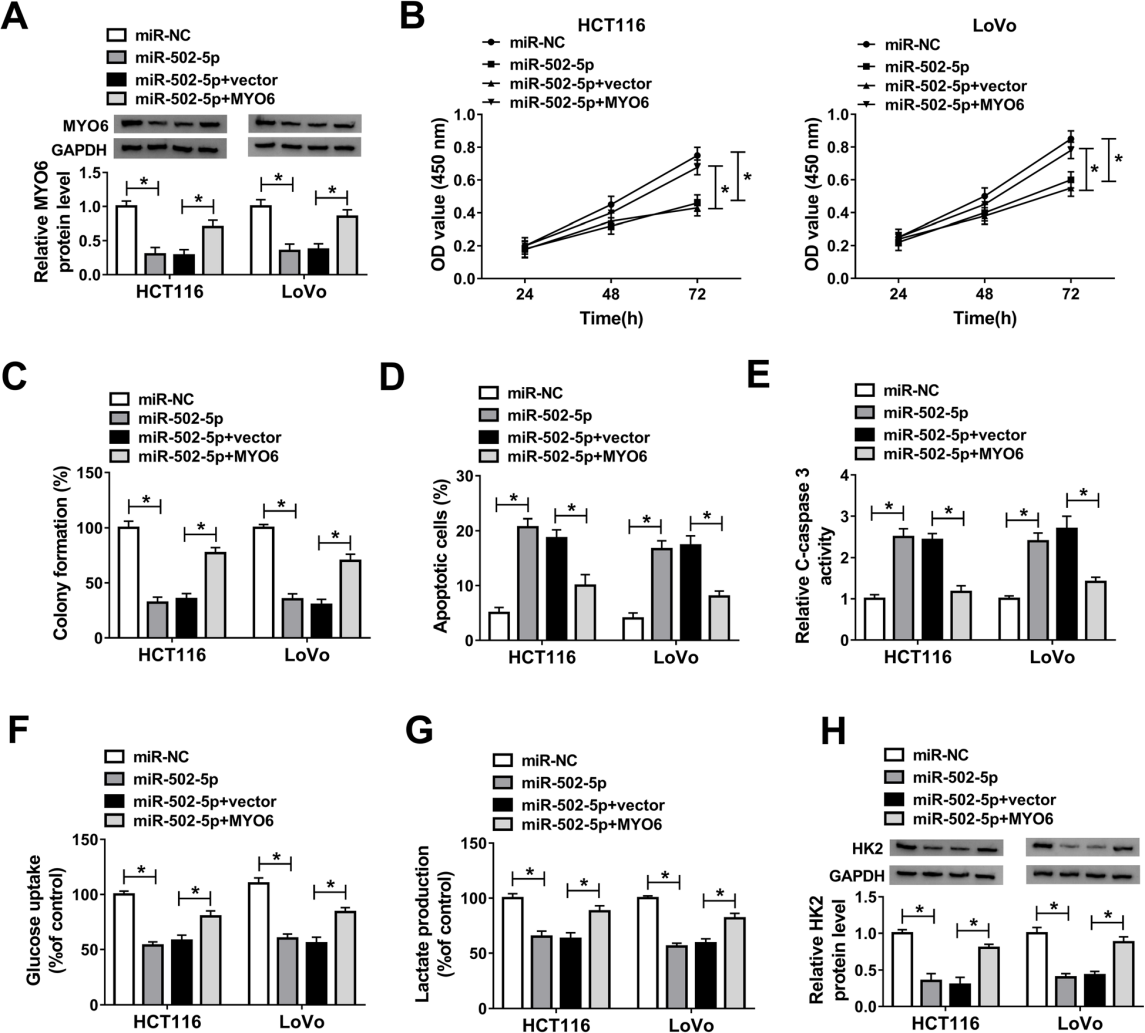
MiR-502-5p regulates the biologic behavior of CRC by binding to MYO6. **a** MYO6 attenuated the inhibition effect of miR-502-5p on MYO6 protein expression in HCT116 and LoVo cells. **b** and **c** MYO6 overexpression hindered the inhibition effects of miR-502-5p on cell viability and colony-forming ability in HCT116 and LoVo cells. **d** MYO6 restored the promotion effect of miR-502-5p on cell apoptosis in HCT116 and LoVo cells. **e** MYO6 restored the promotion effect of miR-502-5p mimic on C-caspase-3 activity in HCT116 and LoVo cells. **f** and **g** Enforced MYO6 expression hindered the inhibition effects of miR-502-5p mimic on glucose uptake and lactate production in HCT116 and LoVo cells. **h** MYO6 overexpression hindered the inhibition effect of miR-502-5p on HK2 protein expression in HCT116 and LoVo cells. *P < 0.05

In order to explore the effects between miR-502-5p and MYO6 on CRC development, the effects between miR-502-5p mimic and MYO6 overexpression on MYO6 protein expression were firstly explored. Western blot analysis showed that miR-502-5p mimics dramatically downregulated MYO6 protein expression in HCT116 and LoVo cells, whereas this phenomenon was decreased by MYO6 overexpression (Fig. 6a). Then CCK-8 and colony formation assays showed that cell viability and colony-forming ability were repressed by miR-502-5p mimic in HCT116 and LoVo cells, whereas these effects were hindered by MYO6 overexpression (Fig. 6b and c). Flow cytometry and C-caspase-3 activity assays revealed that the apoptosis rate and C-caspase-3 activity of HCT116 and LoVo cells were promoted, whereas MYO6 overexpression partially attenuated these effects (Fig. 6d and e). Furthermore, transwell invasion and wound-healing assays investigated that cell invasion and migration abilities were suppressed by miR-502-5p mimic, and these inhibition effects were partially restored by MYO6 overexpression in HCT116 and LoVo cells (Supplementary Figure 3A and B). In addition, data showed that the glucose uptake and lactate production of HCT116 and LoVo cells were inhibited by miR-502-5p mimic; however, this phenomenon was decreased by MYO6 overexpression (Fig. 6f and g). Western blot assay showed that HK2 protein expression was restrained by miR-502-5p mimic, whereas MYO6 overexpression hindered this effect (Fig. 6h). These data demonstrated that miR-502-5p impeded glycolysis, cell proliferation, invasion, and migration, whereas promoted cell apoptosis and C-caspase-3 activity by targeting MYO6 in CRC.

### Hsa_circ_0000231 knockdown represses CRC growth in vivo

**Fig. 7:**
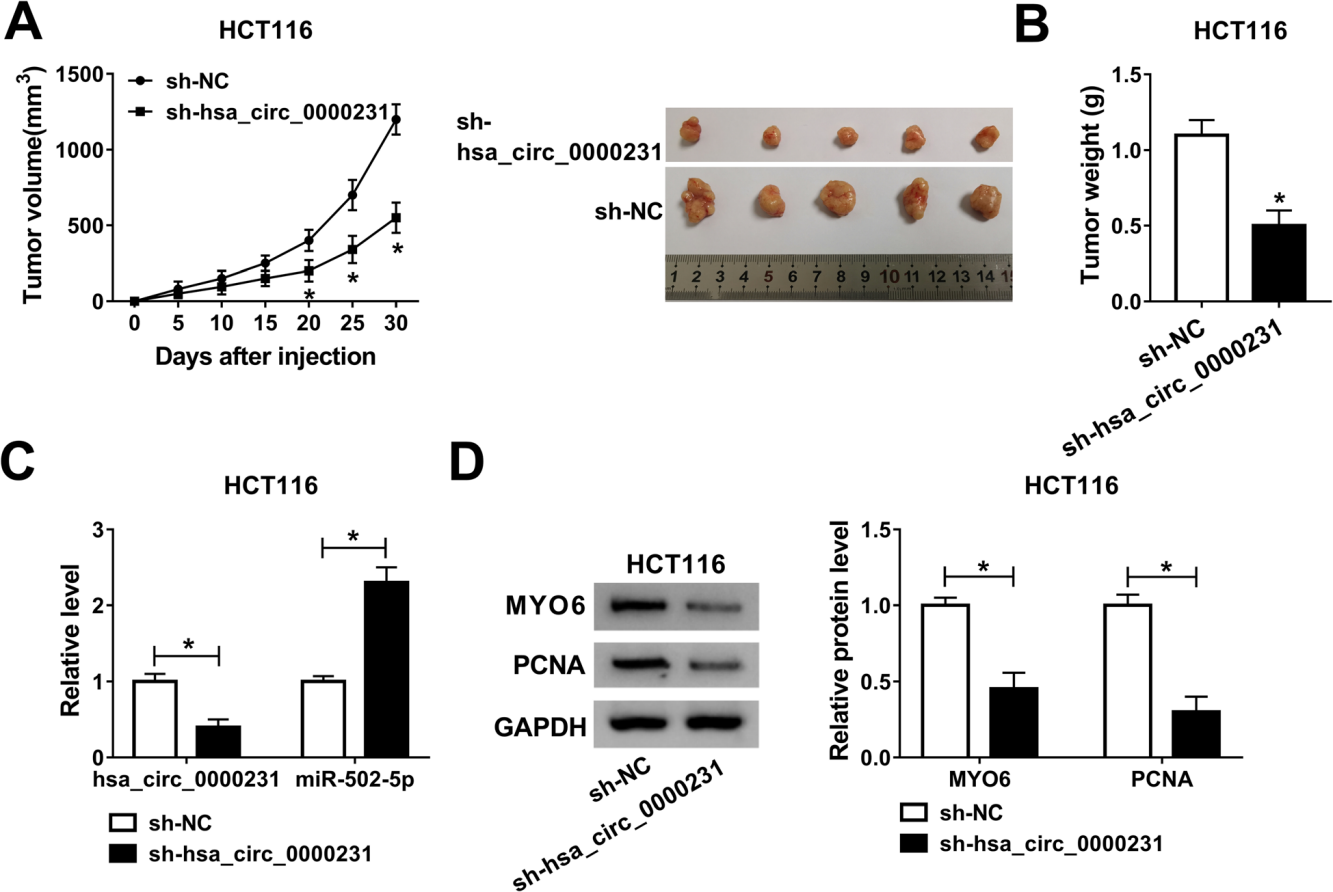
Hsa_circ_0000231 knockdown inhibits tumor growth in vivo. **a** Hsa_circ_0000231 knockdown decreased tumor volume in vivo. **b** Hsa_circ_0000231 knockdown reduced tumor weight in vivo. **c** Hsa_circ_0000231 repression dramatically downregulated hsa_circ_0000231 expression and upregulated miR-502-5p expression in vivo. **d** Hsa_circ_0000231 silencing obviously decreased MYO6 and PCNA protein expression in vivo. *P < 0.05

In order to explore the effects of hsa_circ_0000231 knockdown on CRC growth in vivo, the impacts of sh-hsa_circ_0000231 on tumor volume and weight were firstly detected. Results showed that tumor volume and weight were obviously decreased by hsa_circ_0000231 knockdown (Fig. 7a and b). Subsequently, the effect of hsa_circ_0000231 silencing on miR-502-5p expression was detected, and qRT-PCR results showed that hsa_circ_0000231 knockdown increased miR-502-5p expression (Fig. 7c). Western blot analysis showed that the protein expression levels of MYO6 and PCNA were downregulated after hsa_circ_0000231 repression (Fig. 7d). All these results revealed that hsa_circ_0000231 knockdown inhibited CRC growth by regulating miR-502-5p and MYO6 expression in vivo.

## Discussion

CRC is a prevalent aggressive disease [19, 20]. The metastasis and recurrence of CRC pose an increasing burden on human life. Although much progress has been achieved, the morbidity and mortality of CRC are still high [21]. Therefore, it is necessary to explore efficient or reliable biomarkers in CRC treatment.

CircRNA has been enrolled in the process of CRC. For instances, circ_0026344 was indicated to suppress tumor metastasis in CRC [22]. Zhu et al. explained that circ_0007142 expression was dramatically increased and was related to poor metastasis in CRC [23]. Other like circ_0020397 overexpression contributed to cell viability and invasion, and repressed cell apoptosis in CRC [24]. However, the effects of hsa_circ_0000231 on CRC progression have not been demonstrated. In this study, hsa_circ_0000231 was found for the first time to regulate CRC progression. We found that hsa_circ_0000231 was upregulated in CRC tissues and cells. In order to reveal the role of hsa_circ_0000231 in CRC process, hsa_circ_0000231#1, and hsa_circ_0000231#2 was transfected into cells with control. Results showed that hsa_circ_0000231 knockdown repressed cell proliferation and metastasis, whereas promoted cell apoptosis in CRC. Additionally, hsa_circ_0000231 was indicated to inhibit the glucose uptake and lactate production of HCT116 and LoVo cells. In order to further identify the effects of hsa_circ_0000231 on CRC development, in vivo tumor formation assay was also employed. And data unveiled that hsa_circ_0000231 knockdown decreased tumor volume and reduced tumor weight in CRC in vivo. These evidences indicated hsa_circ_0000231 silencing hindered CRC development and glycolysis.

CircRNA was indicated to regulate gene expression by sponging miRNA [25]. Thus, for further revealing the mechanism of hsa_circ_0000231 in regulating CRC process, the miRNA associated with hsa_circ_0000231 was predicted by the circular RNA interactome online database. Results showed that hsa_circ_0000231 was a sponge of miR-502-5p. It has been illustrated that miR-502-5p was indicated to inhibit cell proliferation and metastasis in bladder cancer and gastric cancer [12, 13]. In breast cancer, miR-502-5p was downregulated and suppressed cell proliferation, whereas promoted cell apoptosis [26]. In this study, miR-502-5p was found to repress cell proliferation, migration, and invasion, whereas promoted cell apoptosis in CRC, which was consistent with previous studies. Besides, we also observed miR-502-5p inhibitor hindered the inhibition effects of hsa_circ_0000231 knockdown on miR-502-5p expression, cell glycolysis, proliferation, migration and invasion, and the promotion effect of that on cell apoptosis in CRC. These evidences implicated that hsa_circ_0000231 regulated CRC process by associating with miR-502-5p.

To further reveal the regulatory mechanism of hsa_circ_0000231 in CRC progression, its downstream was predicted. Results showed that miR-502-5p targeted MYO6. MYO6 has been indicated to promote cell proliferation in lung cancer and prostate cancer [27, 28]. In addition, You et al. explained that MYO6 downregulation inhibited cell growth and induced cell apoptosis in CRC [29]. MYO6 was also reported that its knockdown hindered cell proliferation, migration, and glycolysis in CRC [30]. These data suggested that MYO6 could promote cell proliferation, migration, and glycolysis, whereas repressed cell apoptotic rate in CRC. In our studies, we found that MYO6 hindered the inhibition effects of miR-502-5p on cell proliferation, migration, invasion, and glycolysis, which suggested MYO6 promoted cell proliferation, migration, invasion, and glycolysis in CRC. Our findings were consistent with previous studies. In addition, we also found that MYO6 inhibited cell apoptosis in CRC.

Summary, we found that hsa_circ_0000231 was overexpressed in CRC tissues and cells, and its knockdown inhibited CRC progression in vitro and in vivo. In addition, hsa_circ_0000231 was associated with miR-502-5p. MiR-502-5p was lowly expressed in CRC tissues and cells, and its inhibitor partially attenuated the inhibition effects of hsa_circ_0000231 knockdown on the biologic behavior of CRC. Furthermore, miR-502-5p was identified to bind to MYO6, and MYO6 overexpression could hinder the inhibition effects of miR-502-5p on the biologic process of CRC. These results might provide a new insight to further study the regulatory mechanism of CRC.

## Supplementary information

**Additional file 1.** Supplementary Table 1 Primer sequences and oligonucleotides used in this study.

**Additional file 2. **Supplementary Figure 1 Hsa_circ_0000231 silencing inhibits cell migration and invasion in CRC. (A and B) Hsa_circ_0000231 knockdown suppressed the invasion and migration of HCT116 and LoVo cells. *P<0.05.

**Additional file 3. **Supplementary Figure 2 Hsa_circ_0000231 knockdown represses cell migration and invasion via binding to miR-502-5p in CRC. (A and B) MiR-502-5p inhibitor attenuated the inhibition effects of hsa_circ_0000231 knockdown on the invasion and migration of HCT116 and LoVo cells. *P<0.05.

**Additional file 4. **Supplementary Figure 3 MiR-502-5p suppresses cell migration and invasion via associating with MYO6 in CRC. (A and B) MYO6 attenuated the inhibition effects of miR-502-5p on the invasion and migration of HCT116 and LoVo cells. *P<0.05.

## Data Availability

Data are to be made available on request.

## Abbreviations

CRC: Colorectal cancer
circRNAs: Circular RNAs
MYO6: Myosin VI
qRT-PCR: Quantitative real-time polymerase chain reaction
PCNA: Proliferating cell nuclear antigen
CCK-8: Cell counting kit-8
RIP: RNA immunoprecipitation
miRNAs: MicroRNAs
3′UTR: 3′-untranslated regions
DMEM: Dulbecco’s modified Eagle’s medium
RPMI-1640: Roswell Park Memorial Institute-1640
FBS: Fetal bovine serum
shRNA: Short hairpin RNA
PBS: Phosphate buffer solution
C-caspase-3: Cleaved Caspase-3
HK2: Hexokinase 2
wt: Wide-type
mut: Mutant
