# Supplementary material for: Hsa_circ_0000231 knockdown inhibits the glycolysis and progression of colorectal cancer cells by regulating miR-502-5p/MYO6 axis
Source: World J Surg Oncol. 2020 Sep 29;18:255. doi: 10.1186/s12957-020-02033-0 (PMC7526375; doi:10.1186/s12957-020-02033-0)
Supplement: Supplementary file 1 — Additional file 1. Supplementary Table 1 Primer sequences and oligonucleotides used in this study. [file 12957_2020_2033_MOESM1_ESM.docx]

| Gene | Sequences of primers/oligonucleotides |
| --- | --- |
| si-hsa_circ_0000231#1 | 5’-CCAATGATGACTGGTGGCAAGTCAA-3’ |
| si-hsa_circ_0000231#2 | 5’-GGCGGTGTTGCAGGGTTCATCTTTA-3’ |
| si-NC | 5’-CCAGTAGGTCAGGTGGAACTTACAA-3’ |
| miR-502-5p mimic | 5’-AUCCUUGCUAUCUGGGUGCUA-3’ |
| NC | 5’-UUUGUACUACACAAAAGUACUG-3’ |
| miR-502-5p inhibitor | 5’-UAGCACCCAGAUAGCAAGGAU-3’ |
| anti-NC | 5’-CAGUACUUUUGUGUAGUACAAA-3’ |
| hsa_circ_0000231 sense | 5’-CCGGTCCAGAGTTCTTGGATG-3’ |
| hsa_circ_0000231 anti-sense | 5’-GCTTAGAACAAGGAGATCTACACCA-3’ |
| miR-502-5p sense | 5’- CTCACGATCCTTGCTATC -3’ |
| miR-502-5p anti-sense | 5’-TGGTGTCGTGGAGTCG-3’ |
| MYO6 sense | 5’-GGATCTGTCCGAGCAGGAAG-3’ |
| MYO6 anti-sense | 5’-CTGTACGGGTGAAGCTGGAG-3’ |
| GAPDH sense | 5’-TCTTTTGCGTCGCCAGCC-3’ |
| GAPDH anti-sense | 5’-CCATGGGTGGAATCATATTGGAAC-3’ |
| U6 sense | 5’-CTCGCTTCGGCAGCACA-3’ |
| U6 anti-sense | 5’-CTCGCTTCGGCAGCACA-3’ |

**Supplementary Table 1. Primer sequences and oligonucleotides used in this research**
