# Supplementary figures and images for: Hsa_circ_0000231 knockdown inhibits the glycolysis and progression of colorectal cancer cells by regulating miR-502-5p/MYO6 axis
Source: World J Surg Oncol. 2020 Sep 29;18:255. doi: 10.1186/s12957-020-02033-0 (PMC7526375; doi:10.1186/s12957-020-02033-0)

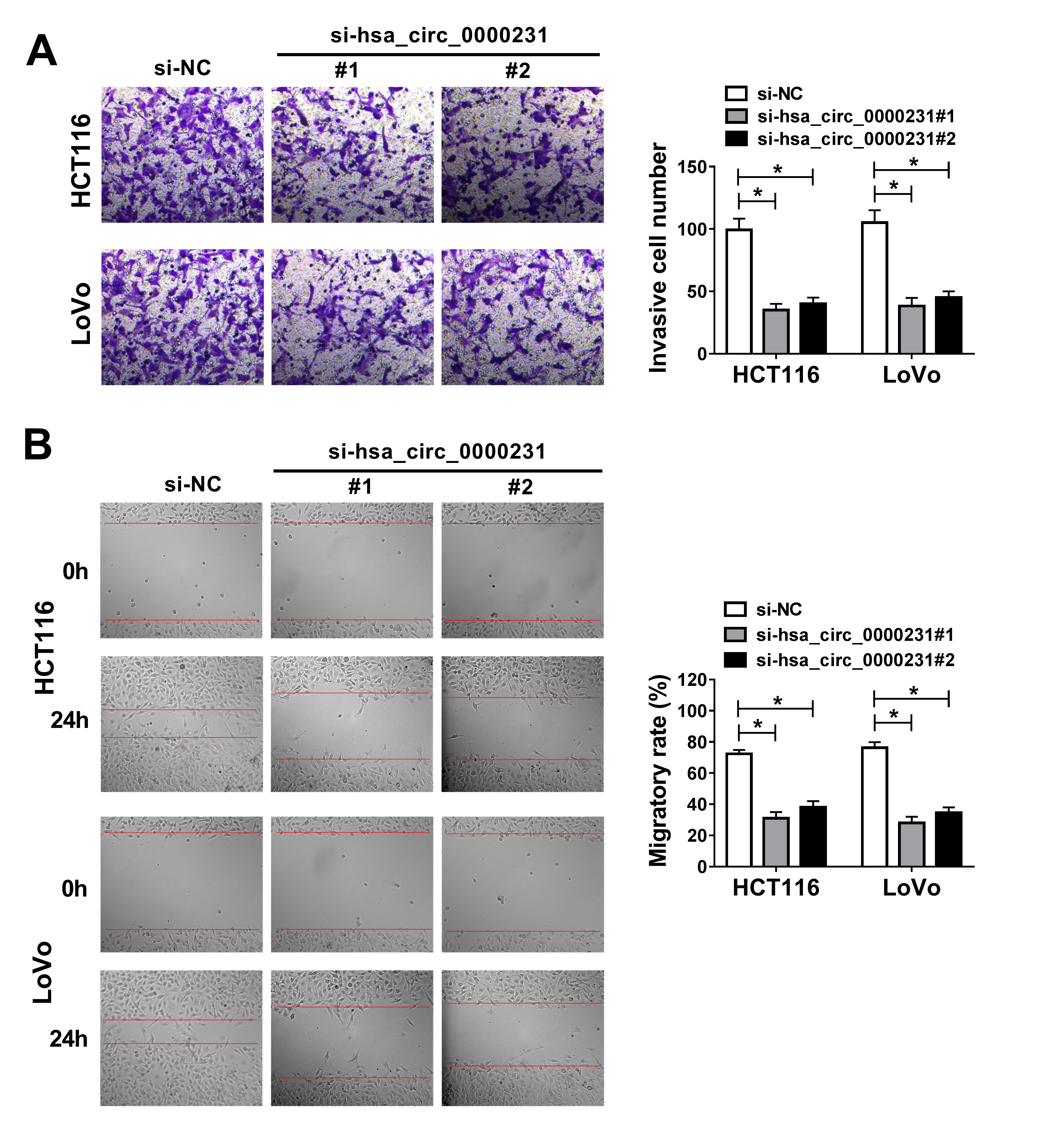

Supplement: Supplementary file 2 — Additional file 2. Supplementary Figure 1 Hsa_circ_0000231 silencing inhibits cell migration and invasion in CRC. (A and B) Hsa_circ_0000231 knockdown suppressed the invasion and migration of HCT116 and LoVo cells. *P<0.05. [file 12957_2020_2033_MOESM2_ESM.jpg]

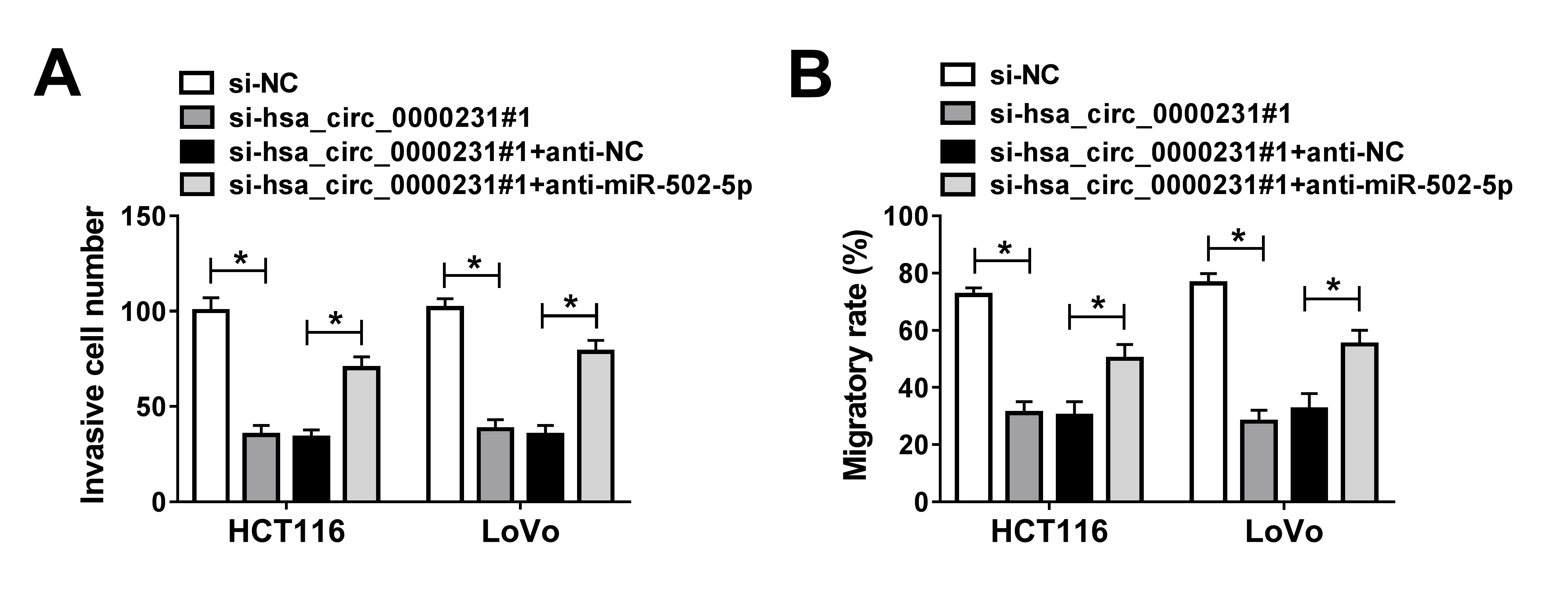

Supplement: Supplementary file 3 — Additional file 3. Supplementary Figure 2 Hsa_circ_0000231 knockdown represses cell migration and invasion via binding to miR-502-5p in CRC. (A and B) MiR-502-5p inhibitor attenuated the inhibition effects of hsa_circ_0000231 knockdown on the invasion and migration of HCT116 and LoVo cells. *P<0.05. [file 12957_2020_2033_MOESM3_ESM.jpg]

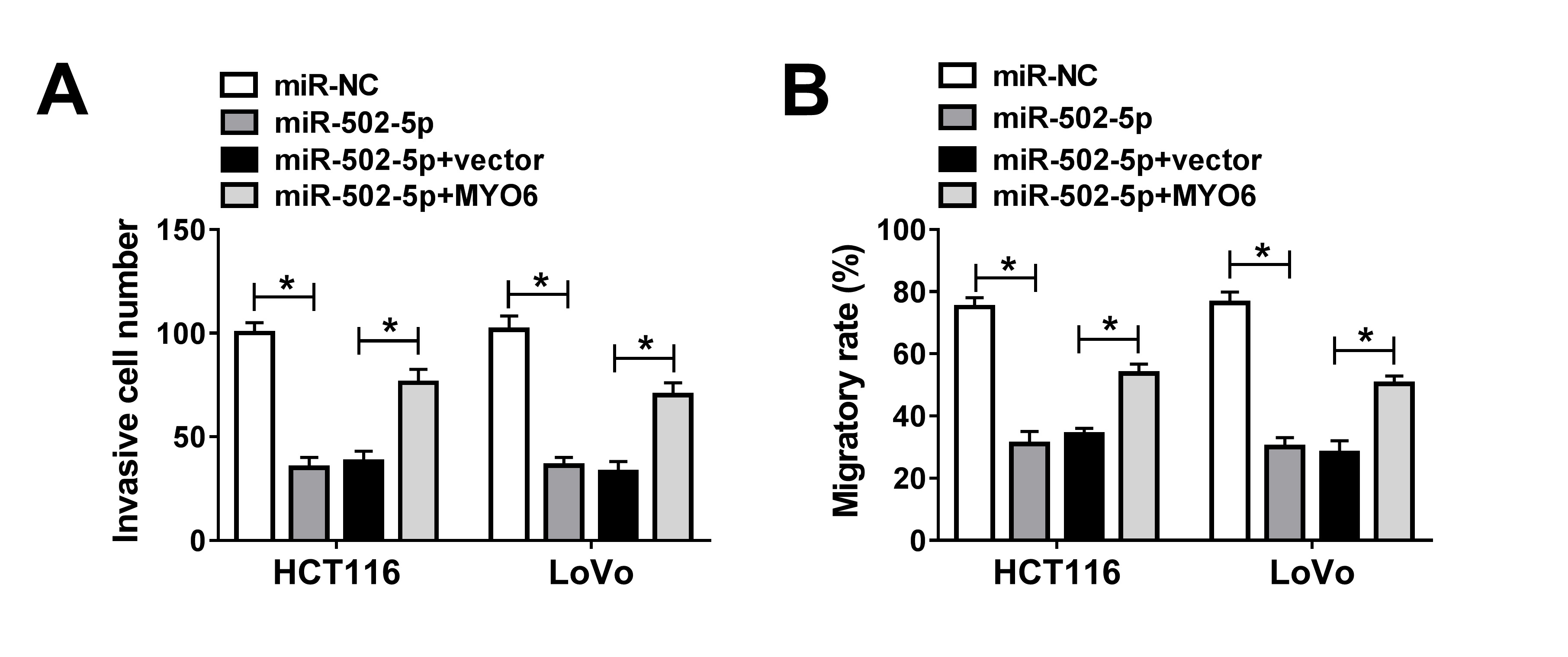

Supplement: Supplementary file 4 — Additional file 4. Supplementary Figure 3 MiR-502-5p suppresses cell migration and invasion via associating with MYO6 in CRC. (A and B) MYO6 attenuated the inhibition effects of miR-502-5p on the invasion and migration of HCT116 and LoVo cells. *P<0.05. [file 12957_2020_2033_MOESM4_ESM.jpg]
